# Using Technology in Pharmacy Education: Pharmacy Student Performance and Perspectives When Visual Aids Are Integrated Into Learning

**DOI:** 10.3389/fphar.2018.01062

**Published:** 2018-09-25

**Authors:** Louise E. Curley, Zimei Wu, Darren Svirskis

**Affiliations:** Faculty of Medical and Health Sciences, School of Pharmacy, The University of Auckland, Auckland, New Zealand

**Keywords:** pharmaceutical science education, pharmacy students, visual learning, technology, disintegration

## Abstract

**Objectives:** The role of the pharmacist has evolved and continues to evolve. The traditional role of the dispenser has been replaced with a patient-centered profession. This requires integration and application of pharmaceutical knowledge and skills to solve patient therapeutic problems and advance patient care. Therefore, having evidence-based teaching strategies for learning within pharmaceutical sciences is essential. New and maturing technologies enable traditional principles of pharmaceutical science to be visualized. We aimed to explore pharmacy students' performance before and after visual aids for learning are integrated within pharmaceutical science teaching. Student's opinions and views of the visual aids were determined.

**Methods:** Students were taught about selected pharmaceutical science concepts at two time points; during the second teaching point, visual aids were introduced. Students' performance was compared before and after the implementation of visual aids using pre and post-quizzes. Alongside the post-quiz an evaluation was also completed by the students; a descriptive analysis was conducted for the Likert-type responses and an in-depth thematic analysis of the student's free-text questions was completed using an iterative process.

**Results:** Significant differences were seen between pre and post-quiz sessions for total score and questions that mapped to the revised-Bloom's taxonomy lower and higher categories. Student evaluation of the visual aids were positive. Interesting themes and subthemes emerged regarding the perspectives of pharmacy students to these visual aids. Students indicated visual aids made it easier to understand, compared to written or verbal explanations, and helped with the application of pharmaceutical science concepts. However, a minority of students reported that the visual aids were irrelevant, or they did not understand them.

**Conclusion:** Students had better performance after the introduction of, and favorable responses to, the visual aids. Visual aids were a beneficial tool in regards to understanding and application of complex concepts. Improvements can be made; tailoring accompanying descriptions and using more repetition.

## Introduction

The role of the pharmacist has evolved and continues to evolve (George et al., 2010). The traditional role of the dispenser has been replaced with a patient-centered profession that requires provision of care as well as advice and in some cases more clinical oriented roles (George et al., 2010). This role requires, more than ever, the integration and application of pharmaceutical knowledge and skills to solve patient therapeutic problems and advance patient care. The Accreditation Council for Pharmaceutical Education (ACPE) regards pharmaceutical education as an essential element to the development of pharmacists (American College of Clinical et al., 2012) and the Center for Advancement of the Pharmacy Education (CAPE) emphasize the importance of pharmaceutical sciences to “solve therapeutic problems and advance patient-centered care” (Medina et al., 2013). Pharmaceutical science is therefore a core component of pharmacy education. The manner in which pharmacy students learn knowledge and skills is of utmost importance, and it is important to reflect on the teaching strategies used to enhance learning within this field. New and maturing technologies enable traditional principles of pharmaceutical science to be visualized (Curley et al., 2017a). Whilst there has been some literature to describe the perspectives of visual teaching on pharmaceutical science learning (Fox et al., 2007; Yellepeddi and Roberson, 2016), limited research is available surrounding effective teaching strategies in pharmaceutical science education and its wide application within pharmacy practice and pharmacy curricula to date (Curley et al., 2017b).

Visual methods for teaching have been shown to help enhance student learning, including improving understanding of complex information. In two publications within this Journal, researchers have presented work that has shown improvements in learning and positive perceptions of visual aids (Fox et al., 2007; Yellepeddi and Roberson, 2016). Fox et al. integrated interactive images of current products to allow students to visually explore the product and the ingredients/labels etc. (Fox et al., 2007). Subsequent online quizzes resulted in increased competency in the year that these images were integrated, and the authors concluded that these images allowed the students to visualize the dosage forms covered during the lectures, and the accompanying quizzes encouraged application of the knowledge (Fox et al., 2007). In a separate research study by Yellepeddi and Roberson, the researchers developed and integrated moderate length (15–20 min duration) videos of the pharmaceutical manufacturing process for different formulations into student lectures (Yellepeddi and Roberson, 2016). Students were asked about their perceptions of these videos and student's summative assessment marks were compared between the year prior to and the years after integration. Positive feedback was received from students, in addition the marks significantly increased in the years after the implementation (Yellepeddi and Roberson, 2016).

The challenge still remains of how to effectively design and develop pharmaceutical science teaching that will facilitate the transfer of theoretical knowledge to clinical practice and allow the application of concepts in a patient-centered manner. The revised-Bloom's taxonomy, reported by Anderson et al. (2001) describes a framework for classifying learning through 6 levels of cognitive processes; remember, understand, apply, analyze, evaluate, and create (Anderson et al., 2001). By using this framework in the design and development of learning and assessment methods, we can evaluate teaching within curricula (Su et al., 2004, 2005).

From 2016, at The University of Auckland in New Zealand, undergraduate pharmacy students are taught pharmaceutical sciences through an integrated curriculum. Students are first introduced to concepts in a foundational module and then move to an integrated-system based approach, where they learn about concepts in the context of, for example, the gastrointestinal system and related conditions. In previous work (Curley et al., 2017a), we have demonstrated the ability to acquire *in-vivo* images of the disintegration of different formulations of paracetamol and have compared them with standard bench-top experiments. We proposed that images, such as these, could be used in the teaching of pharmaceutical sciences to allow for the application of concepts in a patient-centered manner (Curley et al., 2017b). The research in this publication concentrates on evaluating the impact of integrating novel visual aids in the teaching of disintegration principles and associated formulations. Furthermore, this research aimed to explore the perceptions of pharmacy students to these aids, to help facilitate future course design and development within pharmacy curricula.

The first aim of this work was to identify how pharmacy students learning compared after the implementation of the visual aids. To do this we conducted two quizzes (of equal difficulty levels), before and after the implementation of the visual aids (pre and post quizzes). These quizzes centered on the concept of disintegration and associated formulations; specifically we wanted to look at (1) overall scores and distribution, in addition to (2) differentiating lower vs. higher categories of learning, according to the revised-Bloom's taxonomy (Anderson et al., 2001). We also wanted to survey (3) pharmacy students' perspectives on their learning experience after using these visual aids and explore their perceptions, to provide direction for future improvement of student learning.

## Methods

In 2017, the concepts of dissolution, disintegration, and oral dosage forms are taught to the Part II BPharm students at two distinct time points; once as general concepts within a foundation pharmaceutical science module in the first semester and again in the integrated gastrointestinal (GI) system-based module in the second semester.

The foundation pharmaceutical science module in the first semester includes teaching on the concepts of dissolution, disintegration, and oral dosage forms. These concepts are taught in lectures with diagrammatic depictions of the concepts, where appropriate, but have no innovative visual aids (Table 1. details learning outcomes for the pharmaceutical science component of semesters one and two).

**Table 1:** Learning outcomes for the first and second semester pharmaceutical science components of the BPharm in Part II.

**Semester one learning outcomes**
Characterize the properties of the materials as related to states of matter.
Discuss the principles of dosage form design and their application in the development of pharmaceutical formulations.
Recognize the various routes of administration and their role in selection of pharmaceutical dosage forms.
Describe the physical properties and behaviors related to medicinal products such as dissolution, rheology, surface/interfacial phenomena, and relate these to formulation.
Relate the concept of pre-formulation to the process of drug development.
Discuss the biopharmaceutical classification schemes of drugs.
Explain the importance of biopharmaceutics in dosage form design.
Demonstrate the preparatory skills/techniques involved in formulation and dosage form design.
Demonstrate problem solving and self-directed learning skills in the context of pharmaceutical science
Demonstrate information/technological literacy skills in accessing and applying biopharmaceutical information.
**Semester two learning outcomes**
Apply principles of formulation and dosage form design to the choice of drugs/dosage forms, including novel formulation approaches.
Relate the principles of biopharmaceutics to the absorption and pre-systemic metabolism of drugs in the gastrointestinal tract.
Relate principles of medicines management to development of patient-centered plans for the treatment of common gastrointestinal disorders.

Within the integrated GI module in the second semester, an interprofessional faculty drawn from the physiology, pharmacology, chemistry, and pharmacy departments taught a variety of lectures, workshops, and laboratory sessions. However, the pharmaceutical science teaching part is taught solely by pharmaceutical science teaching staff. Disintegration was chosen as a topic to compare between sessions, as it provided a concept that was retaught within a 6 months time frame and is a fundamental pharmaceutical science concept, with visual aids available to accompany the teaching.

Visual aids in the form of Magnetic Resonance Imaging (MRI) images, described in previous work (Curley et al., 2017a), were integrated into two lectures and a corresponding laboratory session; the lectures were “Factors affecting the absorption and bioavailability of medicines following oral administration” and “Formulations for the gastro-intestinal tract” which aimed to develop knowledge of what happens to oral medicines after they are administered to a patient. While the laboratory session focussed on dissolution, the disintegration of a dispersible aspirin tablets is observed in contrast to an enteric-coated aspirin tablet in acidic media that does not disintegrate. Students can directly compare and contrast bench-top experiments with images displaying *in-vivo* disintegration with associated anatomy.

Youtube clips that demonstrated disintegration of conventional, dispersible and effervescent tablets, a raft forming alginate and the *in-vivo* coverage achieved by a range of products designed for local effects in the GI tract were also incorporated into the two lectures: “Factors affecting the absorption and bioavailability of medicines following oral administration” and “Formulations for the gastro-intestinal tract.”

Before and after the integration of the visual aids into teaching, students were asked to participate in research surrounding the use of the visual aids by completing a quiz, ~1 week after the lectures had occurred. At the second time point, students were also asked to complete an evaluation consisting of a Likert-type questionnaire (Table 2) and corresponding free-text questions (Table 3). Participation was voluntary and was administered at the end of a teaching session; students were able to leave and not take part if they chose. Ethics approval was granted by The University of Auckland Human Participants Ethics Committee (019030).

**Table 2:** Description of Likert-type questions from the evaluation of visual aids.

**Likert-type question**
1. The MRI videos and images during the lectures on “Factors affecting the absorption and bioavailability of medicines following oral administration” and “Formulations for the gastro-intestinal tract” helped to develop my knowledge on what happens to oral medicines after they are administered to a patient.
2. The MRI videos and images during the lectures on “Factors affecting the absorption and bioavailability of medicines following oral administration” and “Formulations for the gastro-intestinal tract” helped to develop my knowledge of formulation types.
3. The MRI video clips and images make the learning interesting.
4. The MRI video clips and images at the start of the dissolution laboratory session helped my understanding of the laboratory content.
5. The MRI video clips and images have helped me to apply my knowledge of formulation behavior to its importance in the human body i.e., in patients and their situations.
6. The Youtube videos during the lectures on “Factors affecting the absorption and bioavailability of medicines following oral administration” and “Formulations for the gastro-intestinal tract” helped to develop my knowledge on what happens to oral medicines after they are administered to a patient.
7. The Youtube videos make the learning interesting.
8. The Youtube videos have helped me to apply my knowledge of formulation behavior to its importance in the human body i.e. in patients and their situations.

**Table 3:** Description of the free text questions from the evaluation of visual aids.

	**Free text questions**
9.	What did you find most helpful about using the MRI video clips and images and why?
10.	What did you find least helpful about using the MRI video clips and images and why?
11.	What did you find most helpful about the Youtube videos and why?
12.	What did you find least helpful about the Youtube videos and why?
13.	Do you have any suggestions for improving any of the visual aids in the future?

The quizzes each comprised of 10 questions; 8 which were multi-choice questions with one correct answer and 3 detractors and two short-answer questions, to enable probing of deeper understanding. In the meantime, within each quiz, questions were designed to map to one of five categories of the revised-Bloom's taxonomy; “remembering,” “understanding,” “applying,” “evaluating,” and “creating” (Anderson et al., 2001; Su et al., 2004, 2005). Two questions were developed to map to each category and external advice was sought via a subject librarian who was involved in curriculum development.

Quizzes were distributed by staff not involved in this research; to ensure that students felt no duress to take part in the research. The correct responses to answers in both quizzes were pre-determined prior to the first distribution of quizzes; this ensured there was no bias in the marking of the questions by the researchers. The quizzes were mostly (80%) multi-choice questions, which were marked by the two members of the research team, one from the pharmaceutical science group and the second from the pharmacy practice group. The short-answer questions were also marked by the same two researchers, but then cross-marked for validatory means; any discrepancies in marks were discussed and resolved. When a question wasn't answered this was marked incorrect as it would be for any student assessment.

Quantitative responses to the quiz and evaluation were recorded in Microsoft Excel 2010 (Redmond WA) and crosschecked for accuracy. Questions were grouped into the lower and higher categories of the revised-Bloom's taxonomy; lower scores encompassed questions targeting “remembering,” “understanding,” and “applying” and the higher categories included questions targeting “evaluating” and “creating” (Anderson et al., 2001; Su et al., 2004, 2005). Each student's total score and lower-category and higher-category cumulative scores were transferred to IBM SPSS Statistics version 22.0 (Armonk, NY). The non-parametric Mann-Whitney test (McCrum-Gardner, 2008) was used to test for differences in responses between pre and post-quiz sessions, corrected for multiple comparisons using the false discovery rate (FDR). The Levene's test for homogeneity of variance was conducted to compare the distribution of scores between pre and post-quiz sessions.

Students' evaluation responses were categorized into strongly agree and agree, neutral, disagree and strongly disagree or no response; responses were represented as percentage of total responses. Free-text responses were recorded in Microsoft Excel 2010 and key emergent themes were established following iterative thematic coding. *Two* members of the research team read responses individually, before being coded separately. The two members then discussed the themes and any discrepancies. The codes that emerged were used to identify broad themes and subthemes.

## Results

One hundred and four Bachelor of Pharmacy students were invited to complete a voluntary quiz at two time points during pharmaceutical science teaching sessions during May and October 2017, respectively; this was ~1 week after completion of teaching of the concepts in each semester. The two time points are described as pre- and post-quiz sessions. In the pre-quiz session students were asked only to complete a voluntary quiz, in the post-quiz session students were asked to complete a voluntary quiz and an evaluation of the visual aids used in the course. Sixty-eight (65.4%) respondents completed the pre-quiz and 94 students (90.4%) completed the post-quiz and evaluation.

**Figure 1:**
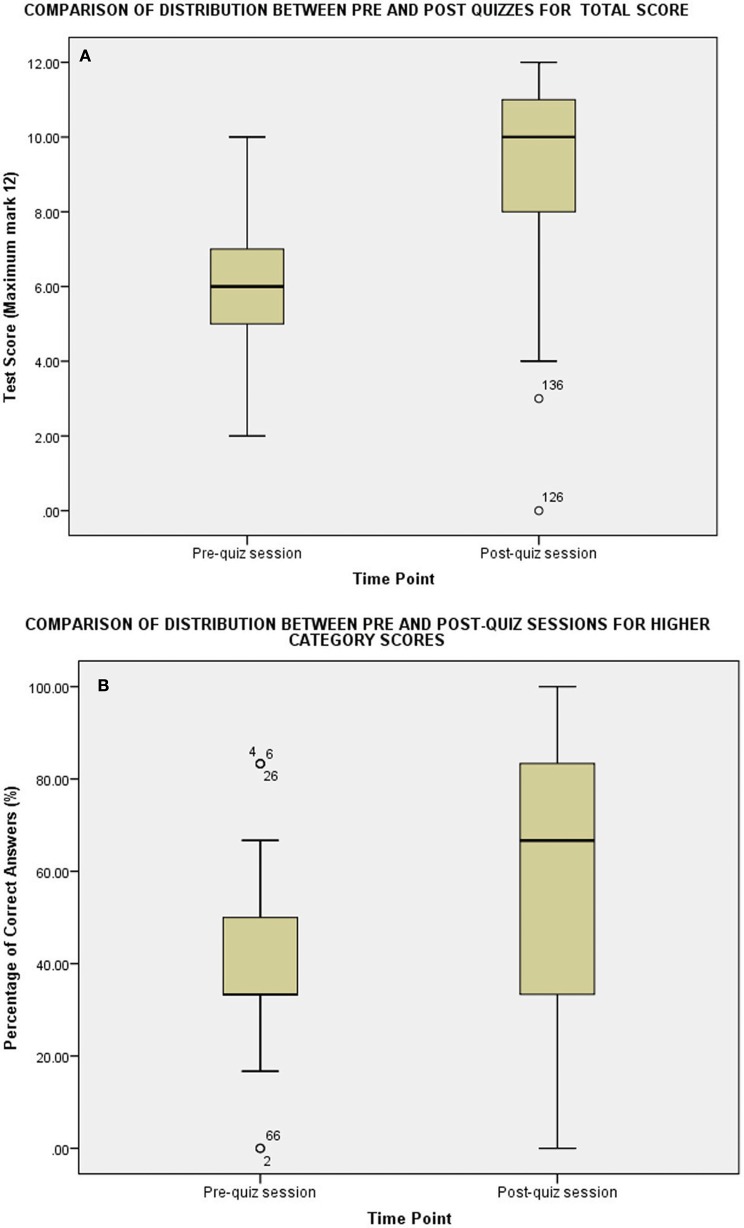
Box and whisker plot to demonstrate the distribution of data over pre- and post-quiz sessions for **(A)** total score and **(B)** the higher-category questions. (pre-quiz *n* = 68 and post-quiz *n* = 94).

The Mann-Whitney non-parametric test was used to assess differences between the pre and post-quiz sessions for (i) total score (ii) lower-category questions (iii) higher category questions. Significance was taken at the False Discovery Rate (FDR)-adjusted *p* ≤ 0.027; an adjusted value was used to account for multiple comparisons. Significant differences were seen between the groups in response all comparisons. The Levene's test was also conducted to see whether the assumption of the same distribution between groups was upheld. These results are all indicated in Table 4. In all three instances, pharmacy students performed to a better degree in the post-quiz session than the pre-quiz session. For total score and the higher-category comparison, the distribution was not just shifted to a higher score, but the overall shape of distribution significantly changed (as indicated in the significant Levene's test of homogeneity of variance) and this is illustrated in Figure 1. The shape of the distribution in both these cases shifted to have a greater proportion of respondents scoring higher.

**Table 4:** Mann-Whitney comparison of scores between pre and post-quiz sessions.

**Comparison**	**Mann-Whitney test**			**Test of homogeneity of variance**^**a**^	
	**Mean rank**	**Mann-Whitney score**	**Significance**	**Levene's statistic**	**Significance**
**Comparison 1: Total score comparison for pre vs. post-quiz sessions**					
Pre-quiz session	43.87	637.000	0.000^**^	4.981	0.027^*^
Post-quiz session	108.72				
**Comparison 2: Lower-category quiz questions comparison for pre vs. post-quiz sessions**					
Pre-quiz session	39.85	363.500	0.000^**^	1.642	0.201
Post-quiz session	111.63				
**Comparison 3: Higher-category quiz questions comparison for pre vs. post-quiz sessions**					
Pre-quiz session	57.33	1552.500	0.000^**^	158.233	0.010^*^
Post-quiz session	98.98				

a *Test of homogeneity of variance using Levene's test. Based on Median and with adjusted degrees of freedom*.

* *Significance taken at the FDR-adjusted p ≤ 0.027 to equate for multiple comparisons; all p-values expressed to 3 decimal places*.

** *Indicates significance taken at FDR-adjusted p ≤ 0.027 to equate for multiple comparisons; all p-values expressed to 3 decimal places*.

Regarding the evaluation component, overall, the majority of students' agreed/strongly agreed (A/SA) with all Likert-type scale statements (Table 5) regarding the use of visual aids in the second semester, albeit one student chose to strongly disagree for all statements. Students were particularly positive about 7 of the 8 questions, which all received over 80% agree/strongly agree responses. The most positive response was to question 3 (89.4% A/SA); “The MRI video clips and images make the learning interesting.” This was closely followed by question 1 (88.3% A/SA); “The MRI videos and images during the lectures on ‘Factors affecting the absorption and bioavailability of medicines following oral administration𠈙 and ‘Formulations for the gastro-intestinal tract’ helped to develop my knowledge on what happens to oral medicines after they are administered to a patient.” Although the results were mainly positive, more neutral responses (20.2% neutral) were given regarding question 4; this question asked students whether “the MRI video clips and images at the start of the dissolution laboratory session helped my understanding of the laboratory content.”

**Table 5:** Percentage of responses to each Likert-type question across all students, including no responses.

	**Likert-type scale response per student group expressed in percentage (%)**			
	**Strongly disagree/Disagree**	**Neutral**	**Strongly agree /Agree**	**No response**
The MRI videos and images during the lectures on “Factors affecting the absorption and bioavailability of medicines following oral administration” and “Formulations for the gastro-intestinal tract” helped to develop my knowledge on what happens to oral medicines after they are administered to a patient	2.1	2.1	88.3	7.4
The MRI videos and images during the lectures on “Factors affecting the absorption and bioavailability of medicines following oral administration” and “Formulations for the gastro-intestinal tract” helped to develop my knowledge of formulation types.	1.1	6.4	85.1	7.4
The MRI video clips and images make the learning interesting.	1.1	2.1	89.4	7.4
The MRI video clips and images at the start of the dissolution laboratory session helped my understanding of the laboratory content.	3.2	20.2	64.9	11.7
The MRI video clips and images have helped me to apply my knowledge of formulation behavior to its importance in the human body, i.e., in patients and their situations.	2.1	4.3	85.1	8.5
The Youtube videos during the lectures on “Factors affecting the absorption and bioavailability of medicines following oral administration” and “Formulations for the gastro-intestinal tract” helped to develop my knowledge on what happens to oral medicines after they are administered to a patient.	1.1	7.4	83.0	8.5
The Youtube videos make the learning interesting.	1.1	4.3	86.2	8.5
The Youtube videos have helped me to apply my knowledge of formulation behavior to its importance in the human body, i.e., in patients and their situations.	1.1	8.5	80.9	9.6

In regards to the open ended questions, six key themes and sixteen sub-themes were identified from the students' responses, as outlined in Table 6 and examples are provided of the theme and subtheme in appendix 1. These themes and sub-themes described below.

**Table 6:** Themes and subthemes emerged from the free text comments.

**Theme**	**Emerged Subtheme**
Learning strategies	Visual learning strategies effective Retention
Application of learning	Application of concepts Realism Connection to patient
Knowledge	Connection with theory Formulation concept knowledge
Understanding	Deepened understanding Difficulty understanding Appropriate explanatory notes needed Relevance to learning
Access to visual aids	More access post-lecture More access to these types of examples within topic More access to these types of examples across topics
Broader implications for learning	Provoked attention Made the learning interesting

The first theme to emerge was “learning” strategies. Many of the students described the visual representations of the formulations both within the anatomy and within the Youtube clips to enhance learning; over the free-text responses there was 43 separate comments regarding the effectiveness of visual learning. A sub-set of these students commented that they were “visual learners” and therefore this type of learning aid was a means to clarifying what was being taught within these topics. Students reported that these visual aids allowed for them to visually understand the concepts being taught rather than reading or listening to the concept described. A number of students indicated that this type of learning also committed the concepts to memory.

The second theme to emerge was “application of learning.” The application of concepts was a strong theme that emerged from the free-text questions in this study. Participants reported that the visual aids allowed for the application of the concepts to be understood. Further to this, some made the connection with patient; students explained that they now could envisage the implications of the pharmaceutical science concepts to patients. The visual aids were also described as making concepts “real.”

“Knowledge” was another theme that emerged. Subthemes of connecting with theory and deepening knowledge of formulation concepts emerged in students' answers. Some students said that the visual aids allowed them to connect to the theory that was being taught. Statements regarding students' development of knowledge through the aids were also made.

The fourth emergent theme was “understanding.” Understanding was a key theme that had four distinct subthemes; deepening understanding, difficulty understanding, appropriate explanatory notes needed, and relevance to learning. Many students (22 comments) reported that the visual aids allowed deepening of understanding, allowing students to visually see the concepts and to understand complex topics. On the contrary, a smaller number of comments were made (3 comments) to state that the visual aids were difficult to understand, others remarked that they only understood the visual aids if they also listened to the corresponding audio explanation. Three students also could not see the relevance of these visual aids.

“Access to visual aids” was another theme to emerge. Many students reported that they would benefit from having continued access for revision purposes to these aids. More access to these types of examples encompasses two subthemes—within the course and across other topics. Within the course, more examples, longer examples and more integrated within the lectures.

Lastly “broader implications for learning” was the final emergent theme. Students reported the benefits of the visual aids also provoked attention and made learning interesting, and through this, were able to understand concepts.

## Discussion

The results of this study demonstrate three major findings; that, (1) overall, the visual aids used in this teaching improved total scores and both the lower and higher categories of questions within the quizzes, (2) that there was a significant change in distribution at both the overall scores and the higher category of learning, whereby more students performed better in the post-quiz and (3) the visual aids were perceived positively by pharmacy students as an addition to the pharmaceutical science teaching within the course, aiding with application of knowledge, with many students reporting that they would like to see more visual aids used across the degree programme. However, there was one question within the evaluation that was rated less favorably and there were a minority of students who found the visual aids irrelevant and had difficulty understanding them.

Significant differences were seen in the scores of the pharmacy students when pre and post-quiz sessions were compared. These significant differences were seen in the total scores and also when the scores were analyzed by grouping the questions within the quizzes by lower and higher categories within the revised-Bloom's taxonomy framework. This is an interesting finding; higher scores across all of these comparisons illustrates that the use of visual aids not only helps retention of concepts, but they also promote deeper understanding (*p* = 0.000). In the pre-quiz students found it difficult to use analysis and creative skills within the topic, providing rudimentary answers to the short-answer questions. However, by the second time point, at the post-quiz session the complexity and sophistication of their answers substantially increased.

Another significant finding is the distribution of overall and higher category scores. This change in distribution is shown in Figure 1. What is seen is an increase in the number of students who are performing better overall, and an increase in the proportion of students who are able to answer the higher category questions correctly, not just a shift to a higher score with the same distribution of marks. One could conclude therefore, that the use of these visual aids provides an opportunity for students to understand the concepts and to be able to apply this knowledge in the clinical context.

There has been a growing body of literature supporting the use of visual aids in education; whilst there hasn't been a huge amount of research in the pharmaceutical sciences domain, there has been two distinct studies by Fox et al. and Yellepeddi and Roberson who have both demonstrated that visual learning techniques improve scores, and rate highly by student evaluations (Fox et al., 2007; Yellepeddi and Roberson, 2016). However, the limitation with the comparisons with student performance in assessments was that it was a different cohort of students compared, in both studies. In our study, we compared students pre and post exposure. When we consider the world that students have grown up in today, it has largely been in the computer-age, where they using visual means as a way of learning, so it is not surprising that by integrating visual aids this improves learning (Fox et al., 2007).

We gathered more information on the student perceptions in the Likert-type questionnaire and the free-text responses. Strong themes emerged from the pharmacy students regarding the use of visual aids in teaching of pharmaceutical sciences; students commented on the learning strategies of visual learning being an effective tool and that these visual aids helped with retention of information. Furthermore, the students reported that these visual aids affected their learning on a broader level, grabbing attention, and making learning interesting. Our results are in line with current pedagogical theory that supports the use of visual aids as a way of capturing student attention and enhancing learning (Reinarz, 1991; Okan, 2003). In addition, our research adds to the literature, as it is evident that through these visual aids, and by mapping with the revised-Bloom's taxonomy, we are able to demonstrate the higher levels of learning that these aids can target. Many students reported that the visual aids allowed for application of information; specifically they built a connection between theory and the patient and that it made the concepts realistic and concrete. This is important from two points of view; firstly it provides evidence that these visual aids can help facilitate the translation of pharmaceutical concepts to the clinical context. Using these visual aids could potentially be an additional teaching tool to provide context to student's learning, in particular as a means of enabling the teaching of pharmaceutical sciences with a patient-centered approach.

Secondly, this also demonstrates the utility of using the revised-Blooms's taxonomy in evaluating learning objectives in healthcare curricula. In a similar manner nursing undergraduate programmes have used this mapping to assess clinical teaching within their teaching (Su et al., 2004, 2005).

However, the responses were not all positive; there was one Likert-type question that received more neutral responses than any other question, and subsequently less positive responses. This question was: “The MRI video clips and images at the start of the dissolution laboratory session helped my understanding of the laboratory content.” In hindsight, we believe that this was a poorly worded question and may have caused confusion. The images were shown in lectures prior to the laboratory session, and not directly in the laboratory itself. Some students may have found it difficult to integrate material from a lecture taught 1 week prior with the laboratory content. Moving forward, the intention is to show these images at the end of the laboratory session, when discussing the results from bench-top experiments, to compare the differences between the formulation's actions within the human body.

In addition, within the free-text responses there was a minority of students who stated the visual aids were irrelevant or difficult to understand, especially if you did not listen to the accompanying audio. These are important points; lecture notes and recordings are available to students, and therefore students could choose not to attend the lectures in person, and instead listen to the lecture recordings in their own time. One of the students reported in one of the free-text comments that “they didn't attend the lecture,” which may partially explain the inability to understand the visual aids provided. However, following a teaching session, students have access to slide and audio recordings and all external links. Potentially within the lectures notes, or links to the Youtube clips, there needs to be a short written explanation (as there would be verbally in the lecture) of how this applies to the current lecture.

It must be acknowledged that there could be other factors that contribute toward the increase in the depth and application of knowledge. As the BPharm curriculum at the University of Auckland is a spiral curricula, this could also be adding to the increase in depth of knowledge. The concept of spiral curriculum was introduced by Jerome Bruner in the mid-twentieth century, and has been documented in both medical and pharmacy education (Rockich-Winston, 2017). Students are introduced to the concepts, which then are revisited at later stages in more and more complexity. However, in the case of our students, whilst repetition of the concepts at the latter stage of the research could have added to understanding, we feel that the free-text comments support the notion that it was the visual aids that contributed to the understanding and application of knowledge in the clinical context. Some students made the immediate connection to the patient, reporting that now understood after the visual aids how this would affect the patient. There is the potential for the results of the second quiz to have been influenced by learnings made from the first quiz. While answers to both quizzes were made available to students, this was a requirement of the ethics approval, these were not made available until after the second quiz thereby minimizing this source of potential bias.

Another suggestion by students in the free-text comments was that there should be repetition of the visual aids, so that students would be able become familiar with the content, and that more visual aids should be used across the degree programme. Repetition of the visual aids within these topics is an easily achievable modification. Introducing the visual aids within the foundational material, and repeating within the integrated module will allow for future research, and be able to demonstrate whether the significant change in distribution of scores would still be seen. Future research could surround the integration of similar visual aids to aid with additional concepts and other modules, to see whether these findings are replicated.

As the pre and post-quizzes were anonymous, we were unable to directly pair the results and perform a non-parametric version of the *t*-test (Wilcoxon signed-rank test) (McCrum-Gardner, 2008). In this light, and reflected in the change in number of student responses in the pre-quiz vs. the post-quiz session (68 vs. 94 students completed the quiz), this data needed to be treated as two independent groups of participants. The result of this is that there is less power in the statistical comparison, which may have led to a higher *p*-value. Since all our results (total score, lower, and higher category) all produced significant results, we do not believe that this has impacted the overall messages that this research reports.

## Conclusion

Visualization technologies can be applied to pharmaceutical science education. Pharmacy students had higher test scores post-quiz and favorable responses to the visual aids being used in pharmaceutical science teaching. Visual aids demonstrated clear and beneficial learning outcomes for the pharmacy students, especially in regards to understanding and application. Interestingly, after the use of visual aids there was a significant increase in test scores in the higher categories, alongside a significant change in the distribution of scores, indicating an increase depth of knowledge by more students. Improvements can always be made; tailoring the accompanying descriptions and using more repetition may improve the understanding for the students who found the visual aids difficult to interpret. In addition, integrating more aids, in this topic and across others, seems to be warranted.

## Author contributions

LC contributed to the initial concept, the design of the research, analysis, and interpretation of the data in addition to the writing and approval of the manuscript. ZW contributed to the design of the research, interpretation of the data in addition to the writing, and approval of the manuscript. DS contributed to the initial concept, the design of the research, interpretation of the data in addition to the writing, and approval of the manuscript.

### Conflict of interest statement

The authors declare that the research was conducted in the absence of any commercial or financial relationships that could be construed as a potential conflict of interest.
